# Communicating risk in prenatal screening: the consequences of Bayesian misapprehension

**DOI:** 10.3389/fpsyg.2014.01272

**Published:** 2014-11-06

**Authors:** Gorka Navarrete, Rut Correia, Dan Froimovitch

**Affiliations:** ^1^Laboratory of Cognitive and Social Neuroscience, Department of Psychology, Universidad Diego Portales, UDP-INECO Foundation Core on NeuroscienceSantiago, Chile; ^2^Faculty of Education, Universidad Diego PortalesSantiago, Chile; ^3^Department of Physiology, University of TorontoToronto, ON, Canada

**Keywords:** Bayesian reasoning, prenatal screening, health policies, risk communication, massive screening

At some point during pregnancy women are typically encouraged to undergo a screening test in order to estimate the likelihood of fetal chromosomal aberrations. While timelines vary, the majority of pregnant women are screened within their first trimester (De Graaf et al., 2002). In the event of a positive test result, an invasive diagnostic assessment is usually recommended, namely amniocentesis or chorionic villus sampling (CVS). The combined test, widely considered to be the most feasible and effective screening procedure, involves an integrated assessment of: maternal age, fetal Nuchal Translucency (NT), maternal serum pregnancy-associated plasma protein A (PAPP-A), and free β human chorionic gonadotropin (β-hCG). This assay is most reliable when performed nearest to the 11th week of gestation (Malone et al., 2005), at which its detection rate and false positive rate for trisomy 21, in optimal conditions, are approximately 95 and 5%, respectively (Nicolaides, 2004). A variety of competing screening techniques are available in the first trimester, and though we focus on the combined test in our example below, the point raised in this article applies to each of them.

A first-trimester screening assay carrying a relatively low false-positive rate might seem a reasonable option for women already considered to be at low risk—the vast majority of the pregnant population. Following such prenatal screening for trisomy 21, most women who test positive for high risk proceed with invasive diagnostic testing. This decision to proceed with invasive testing is typically based on the presence of any evidence of increased risk brought to light by the precursory screening test (Nicolaides, 2004). It is important to note, however, that the proportion of those who advance to invasive diagnostic testing is virtually identical to the false-positive rate of initial screening (Nicolaides, 2004).

Applying trisomy 21 as an example (see Figure 1 for a graphical representation of the numbers), the pregnant women who receive a false positive score in their first-trimester screening (~5%) would subsequently undergo a supplementary invasive diagnostic procedure, such as amniocentesis or CVS. This implies that out of every 100,000 pregnant women initially screened, roughly 5100 test positive, out of which ~5000 cases are actually false positives. The follow-up diagnostic tests are associated with serious procedure-related health risks, including a ~1% increased chance of miscarriage (see Mujezinovic and Alfirevic, 2007 for a systematic review; also, a recent nation-wide 11-year longitudinal study in Denmark established an increased chance of miscarriage of 1.4% and 1.9% linked to amniocentesis and CVS respectively, with CVS growing in its predominance worldwide; Tabor et al., 2009). Thus, at least 50 of the above ~5000 false-positive cases that involve normal fetuses ultimately result in diagnostic procedure-induced miscarriage. Of course with either a higher false-positive rate or a lower disease prevalence, those numbers worsen.

**Figure 1:**
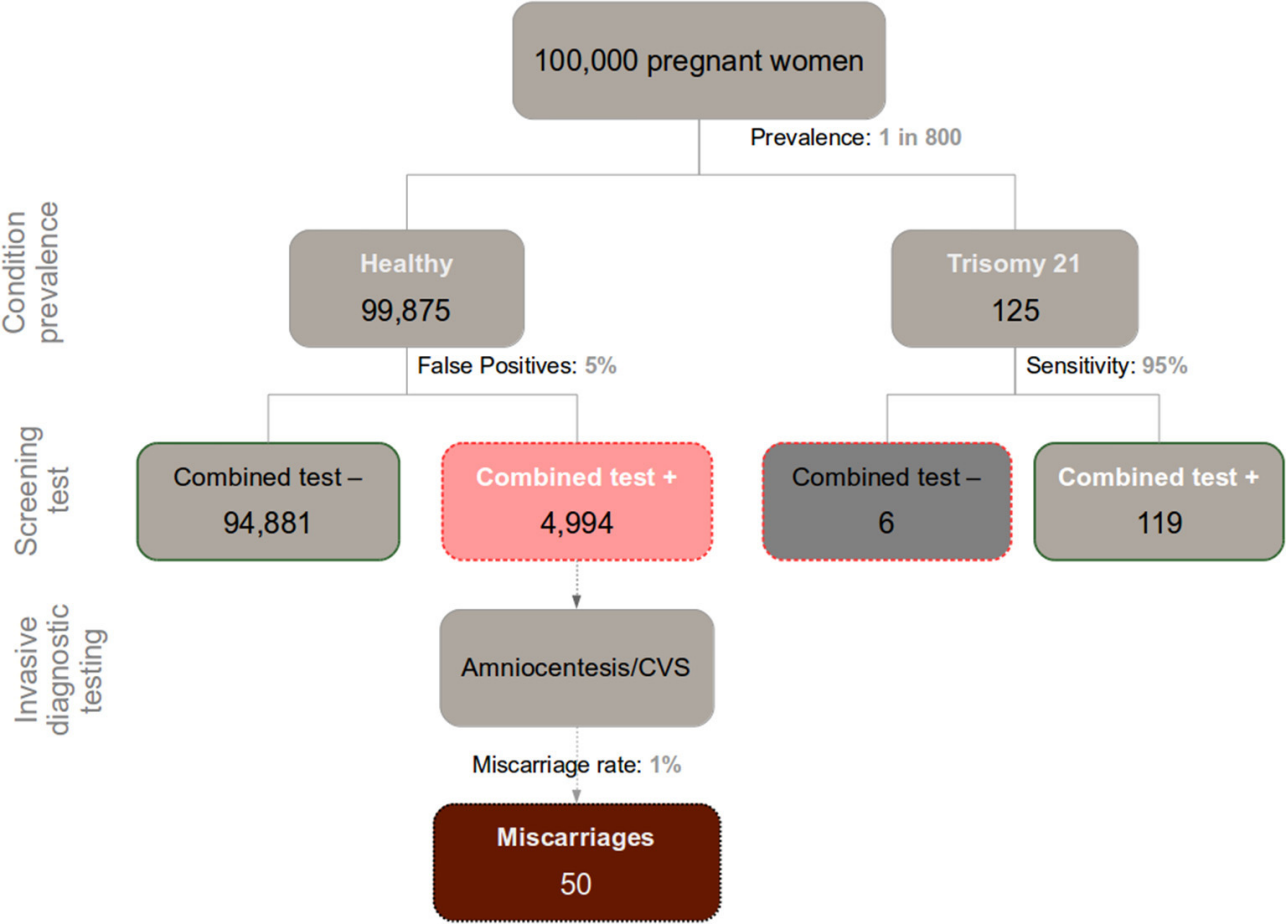
**Chart depicting the relationship between incidence of Down Syndrome (Trisomy 21), false positives in prenatal screening, and miscarriages caused by the recommended follow-up diagnostic assessment (Amniocentesis/CVS) in a sample of 100,000 pregnant women**.

Discerning the trustworthiness of a given positive result in a screening test warrants calculating (typically from the information provided in the respective consent form) the test's positive predictive value (PPV; in this case the proportion of Down syndrome cases relative to the total amount of positive results). This requires knowledge of the base incidence rate of the congenital defect of interest, and the sensitivity and false-positive rate of the test. Computation and proper interpretation of this index, however, is often obscured by the complexity of Bayesian reasoning involved. This, among other factors, may underlie the well-known inadequacy of current procedures intended to achieve informed consent (Green et al., 2004). For 30-year-old pregnant women, the prevalence of Down syndrome is roughly 1 out of every 800 fetuses (Nicolaides, 2004; this statistic varies with maternal age and time-point during pregnancy). In a sample of 100,000 pregnant women of the general population, therefore, around 125 of them would be expected to carry a fetus with the condition. Given the relatively high sensitivity of the screening assay (95% in optimal conditions), a majority of those fetuses are eventually correctly diagnosed with Down Syndrome (~119 out of 125). But when we merge this information with the said ~5000 false positives, we see that 119 positive results in the combined test faithfully reveal trisomy 21, out of a total 5113 (119 + 4994) positive results. Hence, the PPV of the combine test in a screening context nears 2% (119/5113). In other words, there is a 2% chance of actually carrying a fetus with trisomy 21 after testing positive in a screening combined test. This information—essential to an educated decision on the matter—is usually overlooked by practitioners, and generally absent from medical consent forms.

In recent decades, our ineptitude for making sense of Bayesian information has been the subject of extensive study (for a review see Barbey and Sloman, 2007). It is widely recognized that humans struggle in dealing with Bayesian problems presented in terms of normalized probabilities (i.e., relative probabilities or percentages) or in cases of vague information structure (Barbey and Sloman, 2007). A substantial portion of the research on this topic has been done within the scope of medicine and epidemiology, wherein Bayesian inference pervades disease detection and characterization. It is well known that even medical practitioners struggle to interpret such information (Gigerenzer et al., 2007; but see Pighin et al., 2014 for a more optimistic outlook). The issue saliently manifests in the prevailing appeal of massive screening programs to the general public, policy-makers, and physicians alike. This appeal—mainly due to the perceived advantages of early diagnosis—fails to be balanced by sufficient consideration of the high propensity for false alarms and over-diagnosis. The theoretic difficulties that most primary care physicians, for instance, seem to encounter with this type of information (e.g., cancer screening statistics) disposes them to a disproportionate veneration for the potential benefits of disease screening, as they drastically underrate the seriousness of relevant risks.

Gigerenzer et al. have advised on the pernicious use of massive screenings with respect to prostate cancer, HIV infection, etc. (Gigerenzer et al., 2007). False positives can be highly problematic in their ensuing psychosocial turmoil, and with respect to iatrogenic complications and economic costs associated with unnecessary clinical intervention. Moreover the problems, as we have seen above, don't stop at this. Medical knowledge ought to be conveyed lucidly, in a manner that facilitates informed decision-making, specifically accounting for the common cognitive challenges and inter-individual variation observed in probability literacy (Johnson and Tubau, 2013; Lesage et al., 2013; Låg et al., 2014; Sirota et al., 2014a). With respect to clinical screening data, sufficient understanding of the numbers not only entails being in a position to competently evaluate pertinent risks; it further entails being enabled to recognize the possibility that even tests carrying low false-positive rates may simply be inadequate for detecting low-prevalence diseases, particularly in massive-screening settings.

There is growing convergence in cognitive psychology regarding the chief factors that mediate computation of Bayesian reasoning problems. Furthermore some practical improvements in the communication of statistical information have been proposed (while focus on evolutionary underpinnings of these issues appears to have taken a back seat in the literature (Barbey and Sloman, 2007; Navarrete and Santamaría, 2011). With respect to understanding Bayesian problems, apart from intrinsic differences across individuals, in cognitive resources (Lesage et al., 2013; Låg et al., 2014; Sirota et al., 2014a) or numeracy skill (Hill and Brase, 2012; Johnson and Tubau, 2013; Låg et al., 2014), several other factors that pertain to informational presentation *per se* have been deemed relevant to reasoning performance. These include (but are not limited to): problem structure (Barbey and Sloman, 2007; Lesage et al., 2013; Sirota et al., 2014a), the availability of a causal framework (Krynski and Tenenbaum, 2007), representational format (Hoffrage et al., 2002), and reference class (Fiedler et al., 2000; Lesage et al., 2013). Over and above intellectual aptitude, the very manner in which a problem's terms are conveyed to the subject is arguably imperative to the normative Bayesian response.

The above theoretical advancements have translated into numerous helpful strategies for representing and communicating Bayesian information. Regarding medical risk problems, if a subject is provided with the relevant information comprising the standard menu (i.e., hit rate, false positive rate and prevalence; Gigerenzer and Hoffrage, 1995), the most effective way known to facilitate reasoning is to ensure that the problem's set structure is entirely clarified to the subject (Barbey and Sloman, 2007). Natural frequencies (Gigerenzer and Hoffrage, 1995), or more generally, absolute reference classes (Fiedler, 2000; Lesage et al., 2013) are widely considered instrumental to this end. Another important factor, admittedly difficult to disentangle conceptually from the previous one, is computational complexity (Gigerenzer and Hoffrage, 1995; Barbey and Sloman, 2007). Reducing a subject's need to carry out computations (even those of simple arithmetic operations) can substantially enhance reasoning performance. Moreover the use of iconic and interactive representations has been shown to improve performance accuracy (Brase, 2009; Tsai et al., 2011; Micallef et al., 2012; Sirota et al., 2014b). Finally, an increasingly important area of research in this regard pertains to the development of training-programs designed to improve patients' and physicians' comprehension and computation of Bayesian problems (Sedlmeier and Gigerenzer, 2001; Sirota et al., 2014c).

There is a persistent need for advancing research concerning efficacious communication of Bayesian information, such that it can be comprehended by as many individuals as possible—most urgently, those who intervene in health care decision making, such as clinicians and policy-makers. Wide-scale disease screenings hold both advantages and drawbacks (Gigerenzer et al., 2007), and a clear cognizance of their performance characteristics and the numbers underlying them is crucial to the state of public health and safety. At the moment, however, sufficient understanding of them is strikingly scarce, and with each passing year an unacceptable number of prospective parents are pressed to carry out a critical decision of potentially daunting consequences, without adequate knowledge of the important risks. And, of course, the quintessential challenges inherent to Bayesian reasoning are appreciable well beyond the domain of prenatal screening, posing egregious threats to the security and well-being of both the individual and the public.

## Conflict of interest statement

The authors declare that the research was conducted in the absence of any commercial or financial relationships that could be construed as a potential conflict of interest.
